# Correction to: Wig-1 regulates cell cycle arrest and cell death through the p53 targets FAS and 14-3-3s

**DOI:** 10.1038/s41388-023-02601-0

**Published:** 2023-01-27

**Authors:** C. Bersani, L. -D. Xu, A. Vilborg, W. -O. Lui, K. G. Wiman

**Affiliations:** 1 Cancer Center Karolinska (CCK), Department of Oncology-Pathology, Karolinska Institute, Stockholm, Sweden; 2 Present Address: Department of Molecular Biophysics and Biochemistry, BCMM 133, Yale University, New Haven, CT 06510 USA

Correction to: *Oncogene* 10.1038/onc.2013.594, published online 27 January 2014

Supplementary figure 1 was revised, because a beta-actin blot from another experiment was used by mistake when preparing the left panel of the figure.

The CDC42 blot in Suppl. fig 1. belongs to the same blot as that shown in Fig. 1c in the article. CDC42, AKT3, APP and 14-3-3sigma were all analyzed on the same blot. Thus, Wig-1 and beta-actin should be the same in Suppl. fig. 1 and Fig. 1c.

The corrected figure is given below.
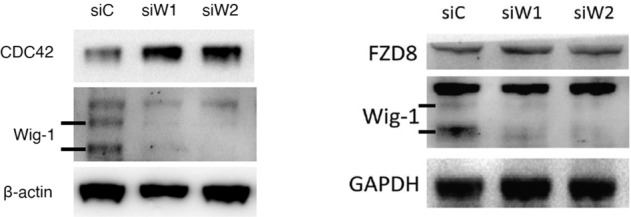


## Supplementary information

Supplementary Figure 1

