# Supplementary material for: Correction to: Wig-1 regulates cell cycle arrest and cell death through the p53 targets FAS and 14-3-3s
Source: Oncogene. 2023 Jan 27;42(9):709. doi: 10.1038/s41388-023-02601-0 (PMC9957718; doi:10.1038/s41388-023-02601-0)
Supplement: Supplementary file 1 — Supplementary Figure 1 [file 41388_2023_2601_MOESM1_ESM.pdf]

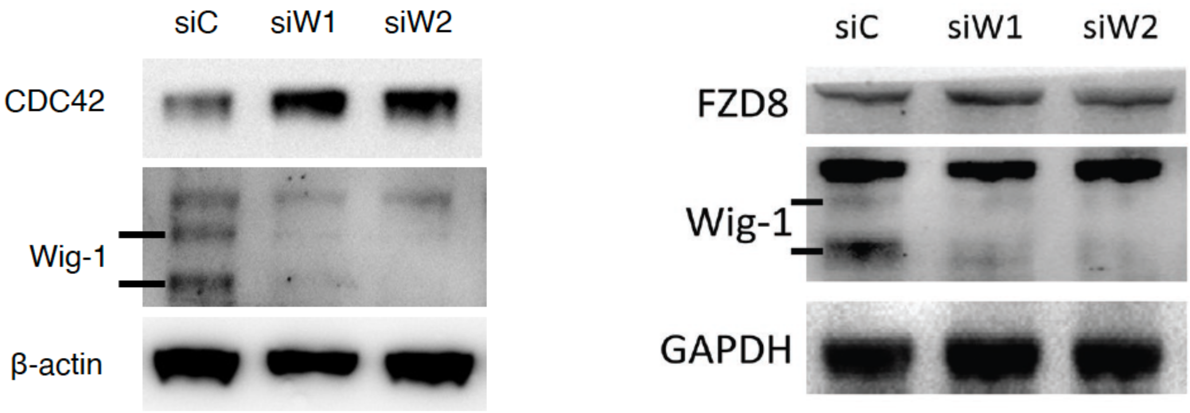

Supplementary Figure 1: Validated Wig-1 targets whose protein expression change after Wig-1 knockdown was inconsistent with the microarray data
